# Supplementary material for: The Role of the Fibronectin Synergy Site for Skin Wound Healing
Source: Cells. 2022 Jul 2;11(13):2100. doi: 10.3390/cells11132100 (PMC9265582; doi:10.3390/cells11132100)
Supplement: Supplementary file 1 [file cells-11-02100-s001.zip › cells-1774106-supplementary.pdf]

Supplementary Figures

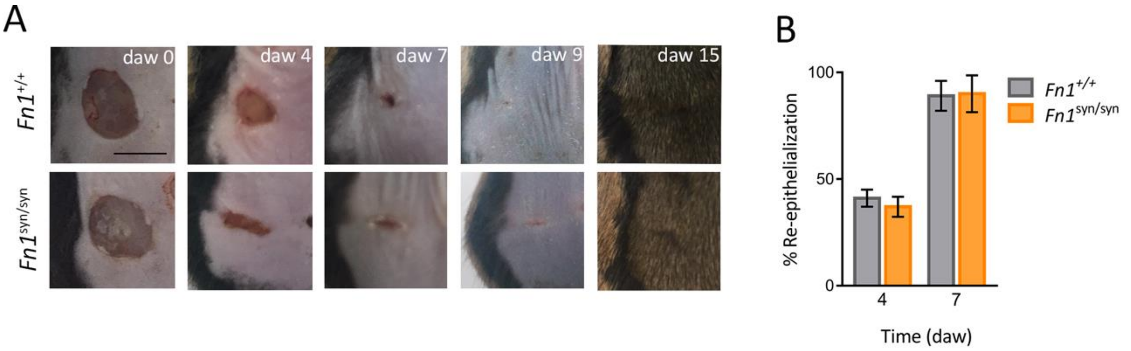

Figure S1. Representative images from *Fn1*$^{+/+}$ and *Fn1*$^{syn/syn}$ mice back wounds at 0, 4, 7, 9 and 15 days after wounding (daw). Scale Bar, 0.5 cm. (B) Percentage of re-epithelialization of *Fn1*$^{+/+}$ and *Fn1*$^{syn/syn}$ wounds.

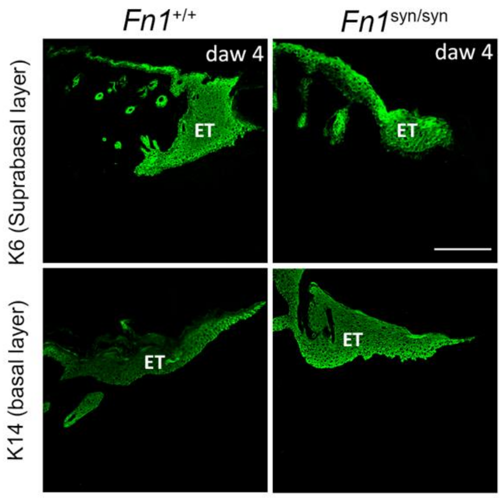

Figure S2. Representative images from Keratin-6 and keratin-14 (green) staining at 4 daw of *Fn1*$^{+/+}$ and *Fn1*$^{syn/syn}$ mice back wounds. Scale bar, 200 μm.

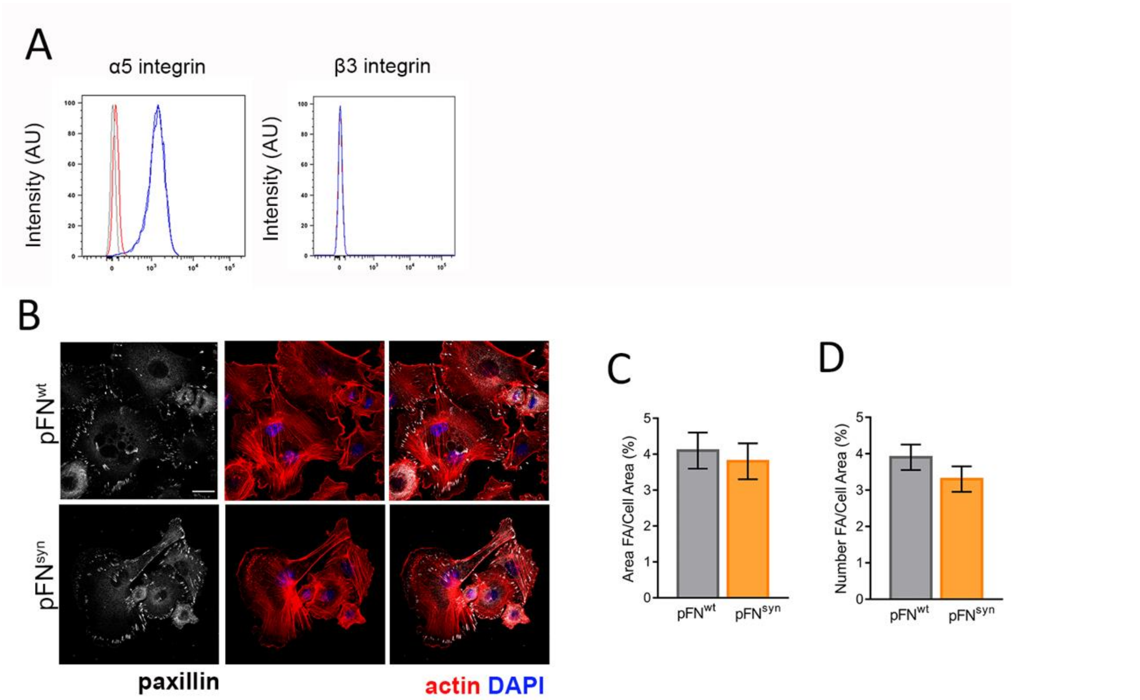

Figure S3. Characterization of keratinocyte cell line. (A) Representative histograms of integrin levels (blue) on keratinocyte surface analyzed by Flow Cytometry. Red line indicates isotype control for α5 or β3 integrin subunit and grey line represents unstained sample. (B) Representative immunofluorescences keratinocytes grown in FN-depleted KGM and seeded on pFN^{wt} and pFN^{syn}. F-actin marked with phalloidin (red), paxillin (white) and DAPI (blue). (C) Quantification of the percentage covered by FA and (D) number of FA per cell area. Between 10 and 12 pictures were analyzed per condition with 1 or 2 cells per picture. Values are shown as mean ± SEM. Statistical significances were calculated using Student's t-test. Scale bar, 10 µm

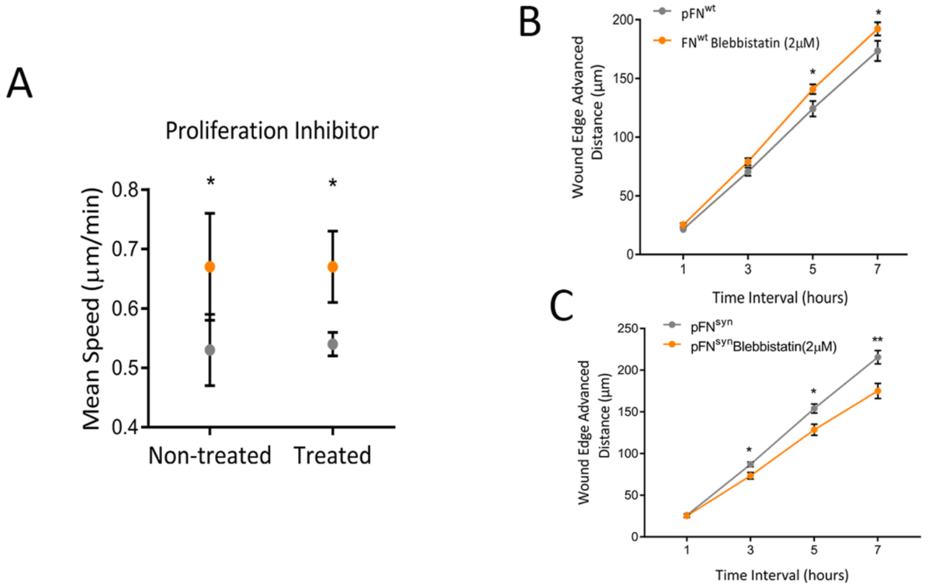

Figure S4. Keratinocyte collective migration. (A) Mean speed of the keratinocyte sheet treated or non-treated with 40 µM Cytosine β-D-Arabinofuranoside to inhibit cell proliferation. (B) Distance

advanced by the cell front in 7 h migrating on pFNwt- and (C) on pFNsyn- coated substrates with or without a treatment with blebbistatin (2µM) to inhibit cytoskeleton contraction. Results are the mean ± SEM from three independent experiments. Statistically significance was determined using the Student's t-test p-value * $p<0.05$ and ** $p<0.01$.
